# Pick’s Disease, Seeding an Answer to the Clinical Diagnosis Conundrum

**DOI:** 10.3390/biomedicines11061646

**Published:** 2023-06-06

**Authors:** Nicole Tamvaka, Sireesha Manne, Naveen Kondru, Owen A. Ross

**Affiliations:** 1 Department of Neuroscience, Mayo Clinic, Jacksonville, FL 32224, USA; 2 Mayo Graduate School, Neuroscience Track, Mayo Clinic, Jacksonville, FL 32224, USA; 3 Department of Clinical Genomics, Mayo Clinic, Jacksonville, FL 32224, USA; 4 Department of Medicine, University College Dublin, D04 V1W8 Dublin, Ireland; 5 Department of Biology, University of North Florida, Jacksonville, FL 32224, USA

**Keywords:** Pick’s disease, primary tauopathy, 3R tau, seed aggregation assays

## Abstract

Pick’s disease (PiD) is a devastating neurodegenerative disease that is characterized by dementia, frontotemporal lobar degeneration, and the aggregation of 3R tau in pathognomonic inclusions known as Pick bodies. The term PiD has adopted many meanings since its conception in 1926, but it is currently used as a strictly neuropathological term, since PiD patients cannot be diagnosed during life. Due to its rarity, PiD remains significantly understudied, and subsequently, the etiology and pathomechanisms of the disease remain to be elucidated. The study of PiD and the preferential 3R tau accumulation that is unique to PiD is imperative in order to expand the current understanding of the disease and inform future studies and therapeutic development, since the lack of intervention strategies for tauopathies remains an unmet need. Yet, the lack of an antemortem diagnostic test for the disease has further complicated the study of PiD. The development of a clinical diagnostic assay for PiD will be a vital step in the study of the disease that will greatly contribute to therapeutic research, clinical trial design and patient recruitment and ultimately improve patient outcomes. Seed aggregation assays have shown great promise for becoming ante mortem clinical diagnostic tools for many proteinopathies, including tauopathies. Future research on adapting and optimizing current seed aggregation assays to successfully detect 3R tau pathogenic forms from PiD samples will be critical in establishing a 3R tau specific seed aggregation assay that can be used for clinical diagnosis and treatment evaluation.

## 1. Pick’s Disease Definition and History

Pick’s disease (PiD) is a rare neurodegenerative disorder clinically characterized by dementia, with frontotemporal degeneration and tau-immunopositive intracellular inclusions known as Pick bodies [1]. As this tauopathy can only be diagnosed postmortem, PiD is a strictly neuropathology term used to characterize cases that present with Pick body pathology and frontotemporal lobe atrophy at autopsy [1].

**Figure 1:**
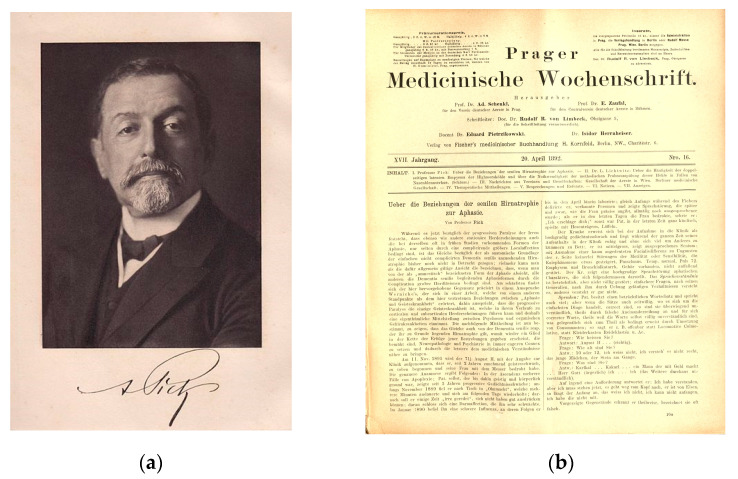
(**a**) Dr. Arnold Pick [accessed from File: Arnold Pick (1851–1924). JPG-Wikimedia Commons] and (**b**) his first paper, titled ‘On the relationship between senile cerebral atrophy and aphasia’, describing the new disease that would later be named after him [2].

In 1892, German physician Dr. Arnold Pick recorded the clinical symptoms of a 71-year-old man, August H, presenting with memory impairment and unusually severe language difficulties [2,3,4,5]. With his inaugural case report ‘On the relationship between senile cerebral atrophy and aphasia’, Pick was not intending to describe a new nosological entity [2,3] (Figure 1). He rather aimed to lay the foundation of what would later become his hypothesis, arguing that dementia was a result of localized brain neurodegeneration and not a diffuse decline of all cognitive abilities and generalized brain atrophy [4,5]. In the years between 1901 and 1906, Pick further refined his hypothesis and published four additional reports, all describing a “new disease” characterized by focal atrophy of the brain regions involved in language and behavior [4,5]. He argued that by examining the affected and unaffected brain regions upon autopsy, one could distinguish between different types of dementia [5]. Pick significantly contributed to the research of dementias with his idea that dementia is a result of the summation of atrophy at different brain regions, a novel concept at a time when dementia was thought to be a diffuse process caused by senility or vascular disease [4,5].

While Pick’s reports were detailing the clinical presentation of patients with some gross neuropathological findings, it wasn’t until 1911 that the neuropathological characteristics of the disease were first visualized [4]. Dr. Alois Alzheimer used the Bielchowski staining method to neuropathologically characterize two dementia cases not fitting the ‘typical’ Alzheimer’s disease (AD) profile and observed what would become the two hallmarks of PiD pathology: argyrophilic inclusions (Pick bodies) and swollen cells (Pick cells) [1,4]. In 1923, Pick’s students (Onari and Spatz) introduced the term “Pick atrophy” to describe cases with atrophy of the frontal and temporal lobes, which quickly became a term used to describe all cases with frontotemporal lobar degeneration, without confirmed presence of Pick bodies [4,6]. Subsequently, when PiD became a separate nosological entity in 1926, the term was used interchangeably for both the clinical and pathological entity, resulting in different interpretations the literature; in fact, the name PiD became almost synonymous to the broader term frontotemporal degeneration [4].

Attesting to that, in 1974, Constantinidis defined three neuropathological subtypes of PiD, of which, only Type A, is consistent with the current definition and criteria for PiD suggesting that during that time presence of pick bodies were not required for a PiD diagnosis. Type B Pick’s disease, as described by Constantinidis, most closely resembles corticobasal degeneration (CBD), as it is characterized by superior frontal and parietal lobe atrophy with ballooned neurons. Lastly, Type C encompasses a wide range of cases with cortical atrophy, presenting with heterogenous clinical syndromes [7].

Around the same time, the scientific community shifted away from the vascular cause of dementia and started focusing on AD neuropathology. Hence, research on PiD became scarce, with only a handful of groups continuing their work on non-AD dementias. Specifically, PiD research was led by two major groups: one in Lund, Sweden, led by Gustafson, Ingvar, and Brun, and the other in Manchester, England, led by Neary, Snowden, and Mann [8]. The two groups came together in 1994 to publish a comprehensive guide of neuropathological and clinical criteria for non-AD cases presenting with atrophy of the frontal and temporal lobes. They were the first to distinguish three subtypes of frontotemporal dementia (FTD); namely, frontal lobe degeneration type, Pick type, and Motor neuron disease type, clearly classifying PiD as a pathology term and a variant of FTD [8].

**Figure 2:**
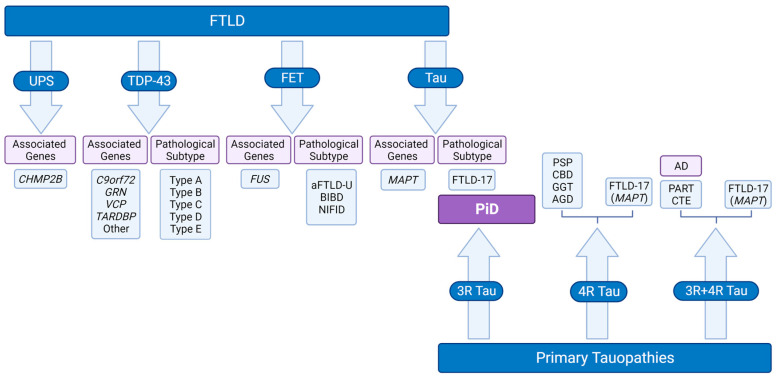
Classification of PiD as a unique neuropathological entity in the intersection of the FTLD and Primary Tauopathy disease spectra. Due to its neuropathological signature, PiD falls under the FTLD spectrum of disease and more specifically the FTLD-Tau clade, as well as the primary tauopathy disease umbrella which is further divided into three categories (3R tauopathy, 4R tauopathy, and 3R+4R tauopathy) based on which form of the tau protein accumulates in the aggregates characterizing each disease. BIBD = basophilic inclusion body disease, NFID = neuronal intermediate filament inclusion disease, FTLD-17 = FTLD-Tau caused by mutations on the tau encoding gene, *MAPT*, on chromosome 17, GGT = globular glial tauopathy, AGD = argyrophilic grain disease, CTE = chronic traumatic encephalopathy. [Figure created via BioRender.com].

Many revisions in terminology have been made since then, which have all culminated in the creation of a Frontotemporal Lobar Degeneration spectrum of disease (FTLD), encompassing all pathological diagnoses of the clinical FTD syndromes [9,10,11]. Important advancements have been made to understand the genetic underpinnings and histopathological signatures of the FTLD spectrum disorders, leading to the classification of FTLD cases based on the composition of the protein inclusions characterizing each disease [12]. Subsequently, all FTLD cases can be further described as FTLD-Tau, FTLD-TDP, FTLD-FET, and FTLD-UPS [12]. PiD is included under the FTLD-Tau pathological subtype of the FTLD spectrum, due to its underlying protein neuropathology (Figure 2).

## 2. PiD as a Unique 3R Tauopathy

PiD is a type of primary tauopathy, along with Progressive Supranuclear Palsy (PSP), Corticobasal Degeneration (CBD) and Primary Age-Related Tauopathy (PART) (Figure 2). Primary tauopathies can be further classified as 3-Repeat (3R; PiD), 4-Repeat (4R; PSP, CBD), and 3R+4R (PART) based on the predominance of the tau isoform present in the cytoplasmic inclusions characterizing each disease (Figure 2) [13]. More specifically, 3R and 4R tau isoforms differ in the presence (4R) or absence (3R) of *MAPT* exon 10 which encodes the second repeat in the microtubule binding domain of the protein [14,15]. In the adult human brain, the 3R and 4R tau isoforms exist in equilibrium; however, in various neurodegenerative diseases we observe preferential increase and accumulation of specific isoforms [14,15]. Interestingly, PiD is the only known primary 3R tauopathy, and due to its rarity, it remains significantly understudied. Thus, the study of PiD has the potential to greatly advance the current scientific understanding of 3R-specific tau pathology as well as informing into the study of other related disorders.

## 3. The PiD Neuropathological Signature

Neuropathologically, PiD is characterized by severe atrophy of the frontal and temporal lobes [1,16]. The atrophy is so excessive that is referred to as “knife-edged” and causes a dramatic decrease in brain weight [1]. Less severe atrophy of the parietal lobe is also usually observed while the occipital lobe, brainstem and cerebellum remain intact [1]. The atrophy extends to subcortical areas including the amygdala, entorhinal cortex, and cingulate gyrus [1,16]. However, the hippocampus remains relatively preserved and the substantia nigra well pigmented, distinguishing PiD from other neurodegenerative disorders [1,16]. PiD brains are also characterized by severe gliosis across the affected cortical areas. Lastly, the white matter of the affected cortical areas becomes granular, and dilation of the ventricles is always observed [1,16].

According to Irwin and colleagues, PiD pathology can be divided into four sequential preliminary phases [17]. Phase I is characterized by moderate to severe tau burden in the frontotemporal neocortex and key limbic structures including the amygdala, entorhinal cortex, cingulate gyrus, dentate gyrus, and subiculum. In Phase II tau pathology extends further into subcortical structures affecting the striatum, thalamus, and locus coeruleus, among others. In Phase III the tau burden extends further into the primary motor cortex and pre-cerebellar nuclei, while in Phase IV, tau pathology is noted in the primary visual cortex [17]. A recent longitudinal neuroimaging study by Whitwell et al. on a small cohort of PiD patients with serial PET imaging described a similar pattern of neurodegeneration starting in the prefrontal and anterior temporal lobe and extending to posterior brain regions with disease progression [18]. Nonetheless, the true sequential staging of PiD pathology in the human brain remains unresolved. There are inherent limitations in studying disease progression from retrospective autopsy series and given the lack of longitudinal data available for pre-symptomatic PiD cases, the progression of Pick’s pathology will not be fully understood, until the disease is able to be clinically diagnosed.

For a PiD diagnosis, certain neuropathological criteria need to be met. These include atrophy of the frontal and temporal lobes and presence of argyrophilic, spherical, neuronal inclusions in the frontal cortex and dentate fascia that are positive upon AT8 (Phospho-Tau Ser202, Thr205) and 3R tau staining, and negative upon 4R tau, 12E8 (Phospho-Tau Ser262), and TDP43 staining.

### 3.1. The Pick Body

**Figure 3:**
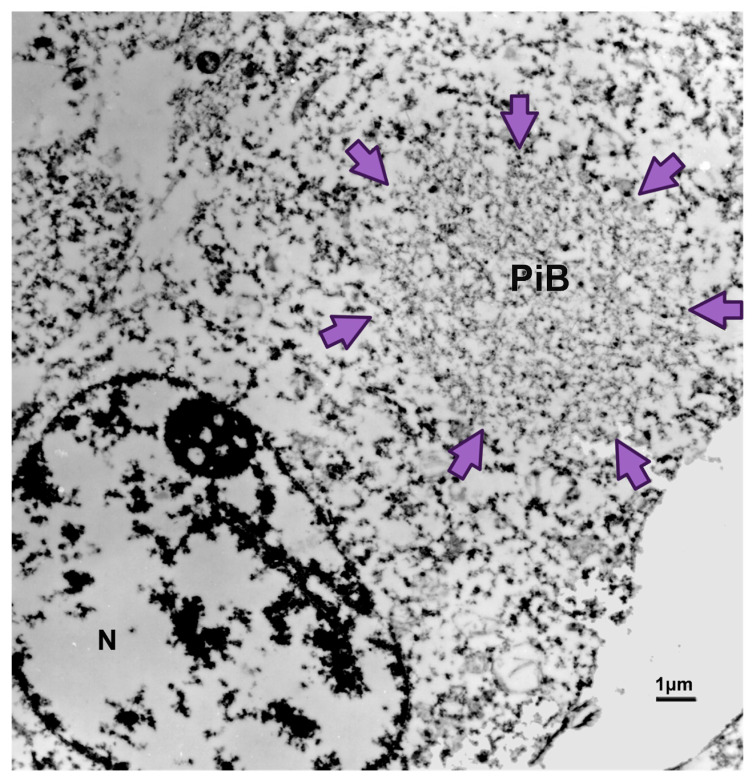
Electron microscopy image of Pick body (PiB) from the dentate fascia of the hippocampus of a PiD brain. Arrows are delimiting the Pick body circumference. N = nucleus. (Courtesy of Dr. Wenlang Lin and Dr. Dennis Dickson).

The hallmark of PiD disease neuropathology is the presence of swollen neurons termed Pick cells and intracellular tau aggregates known as Pick bodies [1,16]. Pick cells are ballooned neurons found throughout the periphery of affected cortical areas. Pick bodies are spherical argyrophilic neuronal cytoplasmic inclusions that are most prominent in the hippocampus but can also be found in layers II and III of the cortex (Figure 3) [1]. They can vary in size depending on the neuronal volume. In most cases, Pick bodies are single, with the exception of the locus ceruleus, where more than one Pick body has been known to appear in the same cell [1]. Pick bodies are negative for α-synuclein, which readily differentiates them from Lewy bodies, and morphologically distinct from other tau aggregates seen in disease conditions, like neurofibrillary tangles in Alzheimer’s disease (AD) [1]. However, little is known about the exact composition of Pick bodies.

A few earlier studies have additionally reported chromogranin A, ubiquitin, oxidative stress marker heme oxygenase, and advanced glycation end-products as components of Pick bodies; however, these observations have not been investigated further [1,19,20,21,22,23]. Recent findings suggest that tau aggregates in vitro and in vivo models of AD are comprised of multiple small nuclear and nucleolar RNAs as well as nuclear speckle components [24]. The idea of protein aggregates mis-localizing cellular components in disease states in is not unique to tau aggregation. In fact, work by Shahmoradian and colleagues suggested a complex composition of Lewy bodies (seen in Lewy body dementia; LBD and Parkinson’s disease; PD) that includes multiple dysmorphic organelles and lipid membranes in addition to α-synuclein [25]. Hence, it can be hypothesized that Pick bodies are also composed of a variety of other proteins, cellular components and miscellaneous RNAs that could play a role in disease mechanisms. Pick body composition, along with Pick body clearance, formation and morphological changes during the disease time course remain elusive and require in-depth investigation.

### 3.2. Tau Structure in PiD

Tau filaments across different diseases are characterized by unique, disease-specific folds. Tau folds are known to be composed of an ordered core, created predominantly by the repeats that make up the microtubule binding domain located at the C-terminus of the protein, with the N- and C-terminal regions forming a coat around the central, ordered core [26,27]. Recently, Falcon and colleagues published the Cryo-EM structure of tau filaments in Pick bodies, significantly advancing our understanding of the unique tau fold present in PiD [27]. More specifically, they identified the Pick core structure seen in the Pick fold as the sequence of tau protein residues K254-F378. This encompasses the C-terminus of microtubule binding repeats 1, 3, and 4 and the 10 amino acids downstream. They additionally described wide and narrow Pick filaments (WPF and NPF respectively) as the major component of Pick bodies. The NPFs seem to be more common and are composed of one Pick protofilament compared to the WPFs which contain two protofilaments interacting distally. Interestingly, they were also able to show significant differences in the morphology of the elongated, twisted Pick fold compared to the C-shaped fold tau acquires in AD in terms of available phosphorylation sites and selective incorporation of sequences into the ordered core. Better understanding of the tau Pick fold is essential for discovering tau binding partners, identifying draggable targets, and elucidating potential protein–protein interaction within the Pick body.

## 4. Clinical Presentation of a Pathological Disorder

PiD patients will most frequently present with symptoms consistent with two clinical syndromes: behavioral variant frontotemporal dementia (bvFTD) or primary progressive aphasia (PPA) [1,17,18,28]. bvFTD is clinically characterized by executive dysfunction, lack of empathy, and disinhibition, while PPA is defined by apraxia of speech, agrammatism, and overall speech deficits [29]. A recent publication by Choudhury and colleagues describing the clinical and pathological characteristics of 21 PiD cases reported a median age at disease onset of 59 years for patients with symptoms consistent with bvFTD, while patients with PPA had an earlier age at onset, with a median of 52 years of age [18,28]. The overall reported median age at onset was 54 years, and the median disease duration was 10 years, without significant differences between the two phenotypes. These observations aligned with a previous report of an independent PiD cohort composed of cases presenting mainly with bvFTD reporting mean age at onset 57.0 ± 12.5 years and mean disease duration 8.6 ± 3.9 years [17]. Overlap of the two syndromes is not uncommon in PiD patients. In fact, Choudhury et al. further observed that 57% (*n* = 12) of cases initially presented with bvFTD and 33% (*n* = 7) with PPA variant [28]. Of those cases presenting with bvFTD, 41% developed PPA as a secondary syndrome. Similarly, 42% of cases with PPA as primary syndrome, developed bvFTD with disease progression.

While FTD and PPA diagnoses are the most frequent among PiD patients, AD, corticobasal syndrome (CBS) and amnestic dementia diagnoses are also common [17,28]. Interestingly, the prominent behavioral changes observed in PiD patients early on in disease progression raises the concern of patients being misdiagnosed with neuropsychiatric conditions and never being seen in a dementia clinic or receiving the right care. In fact, there is great overlap between bvFTD symptoms and the symptoms of various psychiatric disorders. In fact, there are earlier reports of bvFTD patients being diagnosed with obsessive-compulsive disorder (OCD), schizophrenia, and bipolar disorder in the early stages of the disease [30,31,32,33]. Lastly, bvFTD and PPA are both syndromes associated with the entirety of the FTLD spectrum and primary tauopathies PSP and CBD, making a possible clinical diagnosis of PiD based on symptomology extremely challenging [13,29].

The high percentage of differential diagnosis for PiD also complicates the definition of a positive family history for the disease. PiD is considered to be a sporadic disorder, with only a small number of rare *MAPT* mutations having been described in patients with FTD and Pick’s pathology, discussed below. However, very few of these reports define the status of a positive family history. Furthermore, the strong association of PiD with bvFTD and an early stage clinical presentation with neuropsychiatric symptoms suggests the need to establish a PiD-specific definition of a “positive family history for PiD”. Typically, the presence of family history for neurodegenerative disorders is defined by the existence of dementia or parkinsonism in the patient’s family. Whether that definition should be expanded in PiD and other diseases clinically presenting as bvFTD to include a family diagnosis of neuropsychiatric conditions as well remains to be seen.

## 5. The Need for an Ante Mortem PiD Diagnostic Test

Over the last twenty years, there has been very limited research on PiD, and subsequently, the causes underlying disease pathogenesis and susceptibility remain poorly understood. A few research studies have used a small number of PiD samples as a comparison to AD and other 4R tauopathies due to its unique nature as a 3R tauopathy. There has also been work looking at *MAPT* mutations in patients with Pick-like pathology; but with limited understanding of disease pathobiology comes little progress in terms of therapeutic development.

As is the case with many neurogenerative diseases, PiD has no cure. However, the inability to diagnose PiD in life adds another layer of difficulty to fully understanding PiD pathogenesis, advancing research and devising PiD-specific treatments and clinical trials. Without a robust PiD clinical diagnostic test, we cannot design clinical trials, recruit participants, or establish effective PiD treatments. Together with the challenge of differential diagnoses, the inability to diagnose PiD in life has severely limited the amount of PiD longitudinal biospecimens (CSF, blood, plasma) and imaging studies (MRI, PET) available for researchers, influencing our ability to create a clinical PiD cohort, nominate biomarkers for disease progression, or postulate a definitive neuropathological signature of the disease. Subsequently, many aspects of PiD, including patterns of early-stage tau deposition and regional selective vulnerability, remain to be elucidated. Hence, it is imperative to design a safe, easy, and quick clinical diagnostic test for PiD in hopes of making the first step towards a potential PiD treatment.

Over the past decade, tremendous progress has been made in the field of seeding amplification assays (SAA), and their usage for the clinical diagnosis of human prion diseases. Additionally, significant advances have been made in designing non-invasive SAA for α-synuclein aggregation, which hold great potential for becoming the first clinical diagnostic tools for a variety of synucleinopathies. Could that also be the way forward for a PiD ante mortem diagnosis?

## 6. Protein Aggregation Disorders and Seeding Assays

SAA were first developed to improve the understanding and assist in diagnosis of prion diseases, a group of infectious diseases otherwise known as transmissible spongiform encephalopathies (TSEs). TSEs are characterized by misfolded forms of the prion protein, spongiform changes in the central nervous system (CNS), progressive neurodegeneration and are invariably fatal [34,35]. Example of human prion diseases include Creutzfeldt–Jakob disease (CJD) [34], Kuru [36], variably protease-sensitive prionopathy (VPSPr) [37,38], and Gerstmann–Sträussler–Scheinker disease (GSS) [39]. 

The basis of prion disease pathogenesis lies In the conformational change in the normal, cellular prion protein (PrP^C^) to its deleterious form (PrP^SC^; sc = scrapie) [40]. This post-translational process is thought to occur either spontaneously or upon exposure to prion-contaminated tissue and includes the refolding of the secondary structure of the protein into a β-sheet [41]. For this discovery and his work in understanding prion disease, Dr. Stanley Prusiner was awarded the Nobel Prize in Physiology and Medicine in 1977 [42]. Dr. Prusiner proposed for the first time that a pathogenic form of the prion protein (PrP^SC^) can act as an infectious agent, interacting with native forms of the prion protein (PrP^C^) and using it as a template for replication and further spread throughout a living system, organ, or tissue; a concept known as seeding [40].

**Figure 4:**
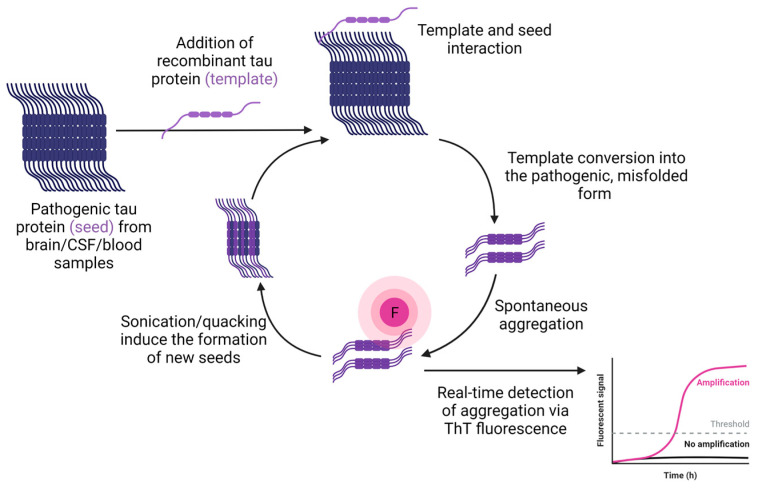
Overview of the seed aggregation assay workflow for tau. Pathogenic tau protein seeds (dark blue) are combined with physiological tau template protein (purple) and their interaction causes the template protein to acquire pathological conformation and from aggregates. This process is measured and quantified in real-time using fluorescent labeling and sonication or quacking methods are used to form new pathogenic seeds. (Figure created via BioRender.com).

For years, the gold standard of prion disease diagnosis has been a post mortem brain autopsy, and a subsequent series of immunoassays for prion protein detection [43]. However, these assays lack the ability to detect low levels of abnormal prion protein and therefore cannot be used in evaluating the presence of prions in accessible biospecimens (blood, CSF, saliva, etc.) of infected humans and animals, which is imperative in understanding the transmission patterns of these diseases [44,45,46]. Thus, SAA have been introduced, with the goal of amplifying ultralow levels of the pathogenic prion protein (seed) to higher levels that can be detected and quantified [47]. SAA can be thought of as being conceptually analogous to the protein equivalent of a PCR for DNA [48], with their goal being to amplify low levels of the pathogenic protein (or seed) to detectable levels that can be measured and quantified. These assays exploit the ability of the pathogenic prion protein to act as a seed, that can interact with healthy protein and induce its oligomerization and conversion into a pathogenic form that will in turn interact with more native protein molecules and induce their misfolding (Figure 4).

One of the first SAA was the protein misfolding cyclic amplification (PMCA) assay introduced in 2001 by Saborio, Permannem and Soto [47]. PMCA consists of multiple cycles of incubation of extremely low levels of PrP^SC^ with high levels PrP^C^ to induce pathogenic conversion, followed by sonication to dissociate the newly formed polymeric aggregates giving them the ability to interact with more PrP^C^ molecules and induce their pathogenic misfolding and aggregation [47]. Caughey and colleagues later developed another SAA known as quacking-induced conversion (QuIC) [49]. QuIC allows the use of recombinant protein, expressed and purified in various cellular systems. This assay substitutes the sonication methods with shaking, minimizes the handling of infectious material and reduced assay time. The addition of Thioflavin T (ThT) fluorescence as a quantifiable readout in real time, further improved the assay giving rise to the real-time QuIC (RT-QuIC). RT-QuIC was introduced in 2010 by Dr. Caughey’s group to assist in the diagnosis and management of prion diseases by providing the ability to detect and quantify prion species in real time [50].

## 7. Seeding Assays as a Tool for Clinical Diagnosis of Neurodegenerative Diseases

Many research groups have formulated the hypothesis that proteins that aggregate in neurodegeneration undergo “prion-like” propagation [51,52,53,54]. Specifically, pathogenic forms of tau and α-synuclein can also act as “seeds” that undergo cell-to-cell transmission in disease-specific patterns within the CNS to induce aggregation [55,56]. Thus, researchers have started leveraging SAA to better define seeding of pathologic α-synuclein in various tissues and biospecimens, aiming to assist in efficient diagnosis and discovery of peripheral biomarkers. The first reported α-synuclein SAA used brain and CSF samples from PD and LBD patients to characterize α-synuclein seeding patterns with great specificity and sensitivity [57,58].

Since then, SAA have undergone many modifications and have expanded to characterize α-synuclein aggregation in MSA, *LRRK2* mutation carriers, and idiopathic rapid eye movement sleep behavior disorder (iRBD), with various levels of sensitivity and utilizing various biospecimens including skin, colon, and olfactory mucosa samples and submandibular glands [57,58,59,60,61,62,63,64,65,66,67,68,69,70,71,72,73]. Most recently, Kluge and colleagues leveraged the SAA to detect α-synuclein aggregation in brain derived extracellular vesicles from blood plasma samples of PD patients [74]. There is currently great interest in shifting away from brain and CSF-based assays and towards detection and characterization of pathogenic protein seeding activity in extracellular vesicles isolated from plasma.

## 8. Seeding Assays for Tau

Compared to α-synuclein, tau SAA are not as well established. The first tau SAA was described shortly after the α-synuclein protocol and aimed to discriminate the seeding profiles of postmortem brain and CSF samples from different tauopathies based on their ability to induce pathogenic conversion of the 3R tau K19CF (CF = cysteine free) substrate [75] (Table 1). The 3R tau K19CF fragment encompasses repeat structures R1, R3, and R4 of the full-length tau protein and is rendered cysteine free to minimize unwanted intramolecular bond formation [75,76]. The assay was successful at detecting and distinguishing the aggregation profile of eight PiD cases compared to PSP, AD, and FTD cases, as well as other non-tauopathy cases and healthy controls. However, the seeding activity of AD samples was almost consistent with the minimal seeding observed in the non-tauopathy cases which contradicts the nature of AD as a mixed tauopathy containing both 3R and 4R tau pathogenic filaments. This could be attributed to the fact that the cysteine free nature of the K19 fragment is not observed in the human body and thus might not fully recapitulate the physiological condition. Additionally, the authors hypothesized that in mixed tauopathies the interactions established between 3R and 4R tau filaments might interfere with preferential seeding of only one of the isoforms [75].

To extend the original assay and establish an AD-specific SAA that can detect both 3R and 4R tau, Kraus and colleagues introduced a point mutation at residue 322 of the AD core (residues 306-378) and tested it as a substrate across a series of pathology confirmed brain samples from different tauopathies [77]. They were able to distinguish the aggregation signal of 16 AD and two CTE brains (both 3R/4R tauopathies) compared to the other diseases. However, the four PART samples included in this study, produced seeding profiles more similar to 4R and 3R tauopathies compared to AD and CTE, attesting to the potential influence of the unique cryo-EM-based structure of tau present in each disease on seeding activity.

Metrick et al. further modified the assay, aiming to propose a single SAA protocol for simultaneous detection of both AD and PiD [78]. To do so, they utilized the 3R Tau K12CF fragment as a substrate, which is comprised of the same tau repeat regions as K19CF, but it is extended by 400 residues on its C-terminus. The new assay was able to successfully discriminate the PiD and AD cases from other tauopathies and non-tauopathy controls. In addition, they were able to discriminate between PiD and AD+CTE samples as the maximum fluorescence reached by the PiD cases was discreetly lower compared to both AD and CTE. To test the efficacy of 3R and 4R full-length tau protein templates, Tennant et al. investigated tau seeding in a range of tauopathies (PiD, PSP, AD, FTLD) and observed optimal sensitivity and detection rates across diseases when they combining the two template proteins in equimolar ratios [79]. Further work has also been carried out on characterizing the aggregation propensity of the AD core and has provided evidence that the AD core is able to spontaneously aggregate in the absence of an inducer [80]. This was also shown to be true for the PiD and the CBD cores but not recombinant full-length tau protein. In addition, Carlomagno et al. used the AD core as substrate for the SAA and was able to successfully detect a distinguishable signal from AD postmortem brain samples compared to CBD, PSP, CTE, and control brains. The AD core was also used in combination with recombinant full length tau protein in a CSF-based SAA to discriminate AD cases from controls [80].

In contrast, little is known about the aggregation propensity and seeding activities of the 4R tau strains that define the 4R tauopathies. Only one study has previously aimed to define 4R tau seeding by utilizing a 4R K18CF tau fragment as a substrate for SAA [81]. Seijo et al. observed distinct amplification signals from PSP and CBD cases as well as from cases harboring tau mutants that favor 4R aggregation (P301L, N279K, and IVS10+3G>A). Attesting to the importance of disease-specific tau conformers and distinct fibril structures within these diseases, the authors were able to classify three different patterns of 4R seeding: (1) tau P301L mutant seeding; (2) CBD and tau N279K mutation seeding; and (3) PSP seeding.

**Table 1:** Summary of studies utilizing seed aggregation assays for pathogenic tau protein detection. fAD = familial Alzheimer’s disease, sAD = sporadic Alzheimer’s disease.

Assay Type	Primary Disease Focus	Samples Tested	Template Protein Type	Publication	Year	Reference
RT-QuIC	PiD	Brain homogenates, CSF	3R tau K19CF	Saijo et al.	2017	[75]
RT-QuIC	fAD, sAD, CTE	Brain homogenates	3R tau K19CF and τ306	Kraus et al.	2019	[77]
RT-QuIC	fAD, sAD, PiD, CTE	Brain homogenates	3R tau K12CF	Metrick et al.	2020	[78]
RT-QuIC	AD	Brain homogenates	2N3R and 2N4R	Tennant et al.	2020	[79]
RT-QuIC	PSP, CBD, FTLD	Brain homogenates, CSF	4R tau K18CFh	Saijo et al.	2020	[81]
RT-QuIC	AD	Brain homogenates, CSF	AD core, PiD core, CBD core	Carlomagno et al.	2021	[80]

While important steps have been made towards the establishment of a 3R tau SAA specific for PiD, several limitations remain. It is important to note that all the PiD experiments described above utilized the same PiD cases. It is thus possible that the observed results could be specific to that small group of samples. In addition, while acknowledging the rarity of the tauopathies highlighted here, especially PiD, the sample sizes included in these studies are relatively small and significant variability is observed across samples of the same group. Therefore, future developments should focus on replicating these assays in larger, independent cohorts to confirm the observed results and to determine the variability across different samples. This will help to establish the utility and reliability of the 3R tau SAA as a diagnostic tool for PiD and other tauopathies, and to inform the development of new therapies for these devastating diseases.

## 9. Conclusions

PiD is a rare neurodegenerative disorder that can only be diagnosed upon brain autopsy based on the presence of its neuropathological hallmarks: 3R tau pick bodies and atrophy of the frontal and temporal lobes. The inability to diagnose PiD in life, in combination with its rarity, has contributed to our lack of understanding of disease risk factors and modifiers and biological processes that contribute to disease pathogenesis or that are dysregulated as a result of 3R tau pathology. Without a deeper understanding of the disease etiology, the development of disease-modifying therapeutics and treatments for PiD will remain unattainable. Without accurate ante mortem diagnosis, the creation of PiD clinical trials or the identification of PiD patients fit for these studies will remain unfeasible. Thus, there is an urgent need for the establishment of a clinical diagnostic test for the diagnosis of PiD. Given the tremendous advancements that have taken place in the world of SAA over the past decade, we believe that they would be excellent candidates for fulfilling this need. Many groups have successfully demonstrated the efficacy, sensitivity, and versatility of the SAA in synucleinopathies and, to a lesser extent, tauopathies, yet the use of PiD samples in these assays has been scarce. The majority of the described assays, however, would still require invasive biopsies that may not be appealing to most patients. As we start thinking about non-invasive methods, blood-derived extracellular vesicles might become the optimal biospecimen for a PiD—specific diagnostic SAA. A better understanding of 3R tau pathology in PiD will improve the current understanding of 3R tau aggregation and its effects on neurodegeneration and cellular dysfunction, as well as inform future studies for other diseases with tau aggregation.

## Data Availability

Not applicable.
